# Atlas of Dental Near-Infrared Transillumination Images

**DOI:** 10.3390/diagnostics14111154

**Published:** 2024-05-30

**Authors:** Nikolaos Angelakopoulos, Clara Isabel Anton Y Otero, Ademir Franco, Lydia Vazquez, Julian Leprince, Marwa Abdelaziz

**Affiliations:** 1 Department of Orthodontics and Dentofacial Orthopaedics, University of Bern, 3012 Bern, Switzerland; 2 Division of Cariology and Endodontology, University Clinics of Dental Medicine (CUMD), University of Geneva, 1211 Geneva, Switzerland; 3 Division of Forensic Dentistry, Faculdade São Leopoldo Mandic, Campinas 13045-755, Brazil; 4 Department of Orofacial Rehabilitation, University Clinics of Dental Medicine (CUMD), University of Geneva, 1211 Geneva, Switzerland

**Keywords:** atlas, DIAGNOcam, dental caries, infrared transillumination, NIRT, forensic odontology

## Abstract

Technological improvements have introduced significant innovations in dentistry and broadened the array of tools and techniques in dental care. One technological development that has been widely researched over the past 20 years is the use of Near-Infrared Transillumination (NIRT) imaging for the diagnosis of dental caries. This paper aims to introduce a comprehensive collection of NIRT images, intended as a reference tool for routine dental examinations, dental research, pedagogical activities, and forensic odontology. The collection presents pairwise clinical and NIRT images categorized as follows: (a) healthy teeth, (b) carious teeth, (c) restored teeth, (d) enamel defects, and (e) diverse findings. This atlas could be a valuable tool for the dental community as it is designed as an identification guide of NIRT illustrated dental features.

Dental caries is the most widespread chronic condition in the world. Despite a decrease in the incidence of significant cavitated dental lesions, most individuals still exhibit early-stage lesions [1]. Traditional methods for caries detection, such as visual examination and dental probes, are adept at identifying clinically visible caries [2]. Although dental X-rays are considered the gold standard diagnostic tool for deep caries and lesions that are clinically concealed, they do not allow early enamel caries diagnosis [3]. Meta-analyses have demonstrated that visual and radiographic examination for the detection of approximal caries has considerably high specificity but low sensitivity, leading to dental practitioners traditionally using a combination of the two methods to obtain the best diagnosis [3,4].

Early identification of carious lesions, acknowledged as a crucial factor for successful treatment, would greatly benefit from novel diagnostic methods that do not expose the patient to ionizing radiation. Near-infrared transillumination and reflectance are non-ionizing imaging modalities that provide information based on the scattering and absorption of near-infrared (NIR) light within dental tissues [5,6,7,8,9]. Both laboratory and clinical research on the use of Near-Infrared Transillumination (NIRT) in the early detection of caries have shown promising outcomes [10,11,12,13,14]. Further developments like automated assistance systems for NILT-based diagnostics have been investigated to increase examiners’ reliability and allow even less experienced examiners to reach accuracies similar or higher to that of specialists [15,16].

An example of the early application of diagnostic imaging is the use of NIRT using the DIAGNOcam™ device (KaVo Dental™, Biberach, Germany). This device uses 780 nm NIRT technology and has an intraoral camera with two flexible extensions: an NIR light that transilluminates the tooth through the periodontal tissues, and a camera that captures images from the occlusal surface of the examined tooth [17]. This technology offers a safe and reliable alternative to ionizing radiation for caries detection in young patients and pregnant women [14,18,19]. Near-infrared transillumination has been described as particularly useful for detecting and monitoring carious lesions in the initial stages and can be integrated into the workflow of risk-based patient management. Numerous publications focusing on diagnostic accuracy, acquisition time, and advantages against radiographic examinations have reported on the importance of implementing NIRT imaging technology in routine dental examinations [14,20,21,22,23]. NIRT, described as particularly useful for detecting and monitoring the initial stages of carious lesions, can be integrated into the workflow of risk-based patient management [19,24,25].

Recent studies and reviews on Near-Infrared Transillumination (NIRT) technology indicate that it could become a valuable alternative to bite-wing radiography for the early detection of proximal caries, particularly in monitoring enamel lesions during recall examinations. Importantly, NIRT does not involve ionizing radiation, allowing for unrestricted frequency of use. Supporting this potential, research has shown that the diagnostic outcomes of NIRT imaging are comparable to those of bite-wing radiographs [14,26]. It was demonstrated that lesions detected in NIRT images closely correlate with X-ray images and clinical assessments (NIRT vs. X-ray image 97%; NIRT vs. clinical 96%) [14,26,27]. 

Furthermore, a recent clinical study found that NIRT devices, when compared to BW radiographs, are more adept at detecting early proximal enamel lesions [28,29]. Additionally, when the NIRT device was used to detect proximal caries with two-year intervals between readings, the reproducibility of caries detection was excellent compared to BWs of the same patients. NIRT appeared to identify more enamel caries lesions than radiographs, revealing approximately four times as many lesions reaching the enamel–dentin junction in comparison to BW radiography [19]. Similar findings have been reported recently [28,30], highlighting the high reliability of NIRT and its strong agreement with clinical and radiographic examinations in detecting dentin caries lesions.

The adoption of this technology by clinicians underscores the necessity for readily available and detailed guidance on interpreting these images. For this reason, we have developed “The Atlas of Dental Near-Infrared Transillumination Images”. 

Our aim with this paper is to raise awareness among dental health professionals regarding the use of NIRT images in daily clinical dentistry by providing a comprehensive atlas covering healthy and carious teeth as well as various NIRT observations of enamel defects and other pathologies. We also aim to highlight the importance of maintaining dental records such as NIRT images for forensic and medico-legal purposes. Potentially, these images could be a great source of ante-mortem data for comparisons in dental human identification.

The atlas presents the most common and relevant findings detected throughout a database of NIRT images. Hence, for didactic purposes, the selected images of dental features were categorized into five classes: (1) healthy teeth, (2) carious teeth, (3) restored teeth, (4) enamel defects, (5) diverse findings. 

The next twenty figures present clinical (on the left) and NIRT (on the right) images presented side by side to build a comprehensive collection of dental images.

## 1. Healthy Teeth

**Figure 1:**
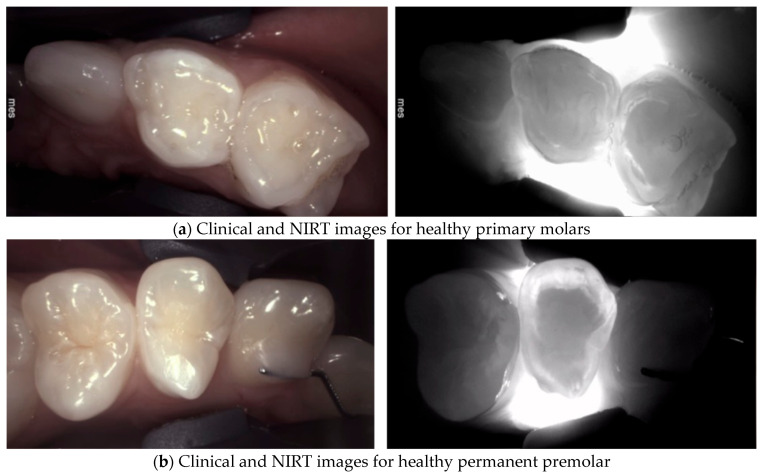
Healthy primary (**a**) and permanent teeth (**b**), exhibit a uniform highly translucent enamel on Near-Infrared Transillumination (NIRT) images. The enamel–dentine junction (EDJ) is visible because of the difference in the light-scattering properties of the enamel and the dentin. Dentin appears as a darker, less translucent central structure due to the increased light scattering related to the lower mineral dentine composition/content [9].

Near-infrared (NIR) light transillumination camera systems operate on the principle that light intensity can be reduced either by absorption, where photons are lost to hard tissue or caries lesions, or by scattering, where the direction of photons changes without a loss of energy. In the NIR light range (700–1500 nm), the wavelengths are significantly longer than those in the visible light range. These longer wavelengths scatter less, allowing deeper penetration into tooth tissue. With NIR light transillumination, each type of tooth tissue (enamel or dentin) is uniformly illuminated. Enamel appears transparent, while dentin scatters light more strongly Figure 1, allowing the two tissues to be distinguished [9,31,32].

## 2. Carious Teeth

**Figure 2:**
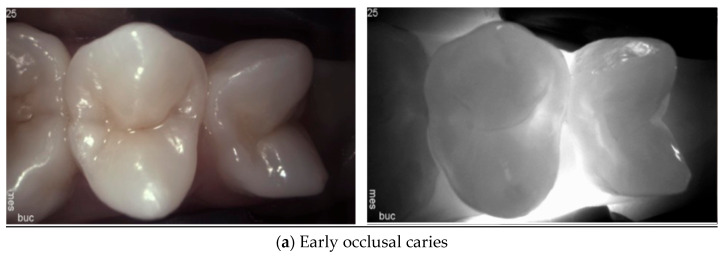

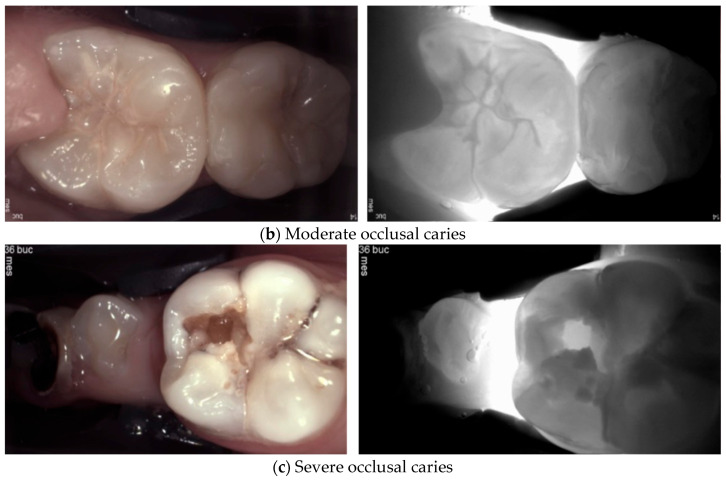
**Occlusal caries**. Early caries (**a**) appear as a thin dark line emphasizing the occlusal fissure system. Moderate caries (**b**) are distinguished as areas of widening of the occlusal fissure system, indicating a wider area of enamel demineralization and the involvement of the EDJ. Severe caries (**c**) present as extensive areas of reduced light transmission and increased scattering. If large cavitation is present, it will be visible as a highly bright and illuminated area (**c**). This is due to the lack of light absorption in the cavitated area when compared to non-cavitated lesions where the light scattering and absorption is increased.

**Figure 3:**
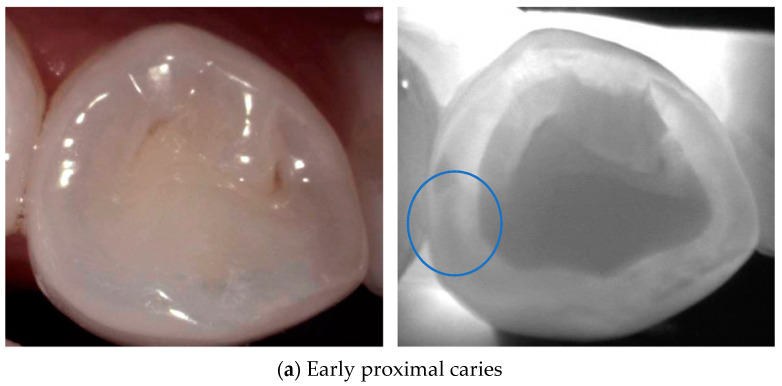

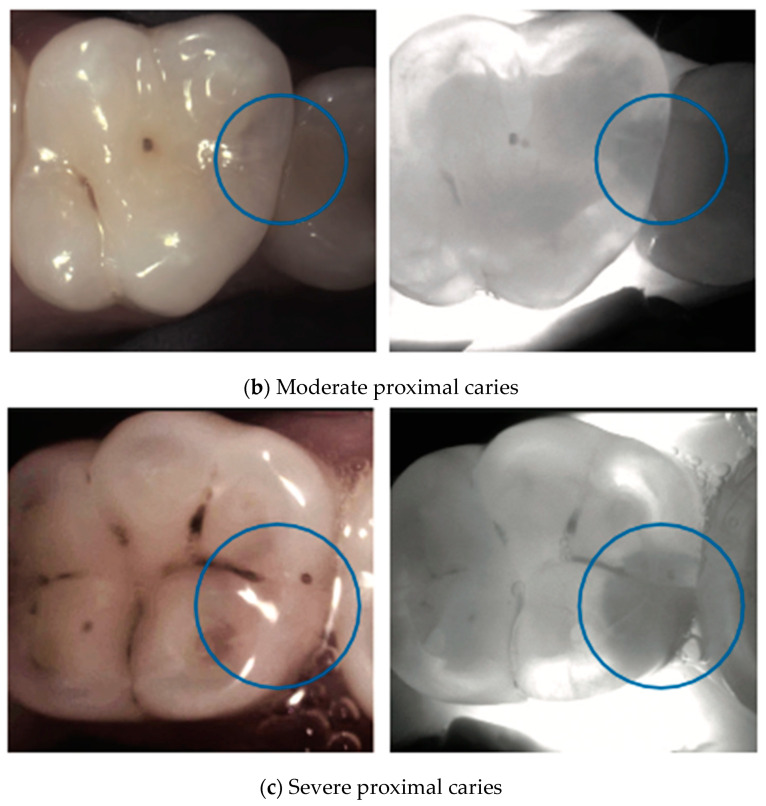
**Proximal caries**. Proximal caries appears as a loss of translucency in the enamel (dark area). Early lesions (**a**) appear as a triangle with the tip pointing toward the enamel–dentin junction, with the progression of the proximal carious lesion into a moderate lesion (**b**), the dark area will expand in the enamel before reaching the EDJ (blue circle). The exact extension of the severe proximal carious (**c**) lesion in dentin (blue circle) is less visible due to the smaller difference in light scattering between sound and demineralized dentin [24].

When tooth tissue is demineralized at the earliest stage of caries, the scattering coefficient increases, resulting in reduced light intensity. This causes caries to appear as a dark area at the marginal ridge for proximal caries or at the occlusal surface for occlusal caries in Figure 2 and Figure 3. The contrast between sound and demineralized enamel is greatest in the NIR range due to the minimal scattering of sound enamel [10,14,33].

## 3. Restored Teeth

**Figure 4:**
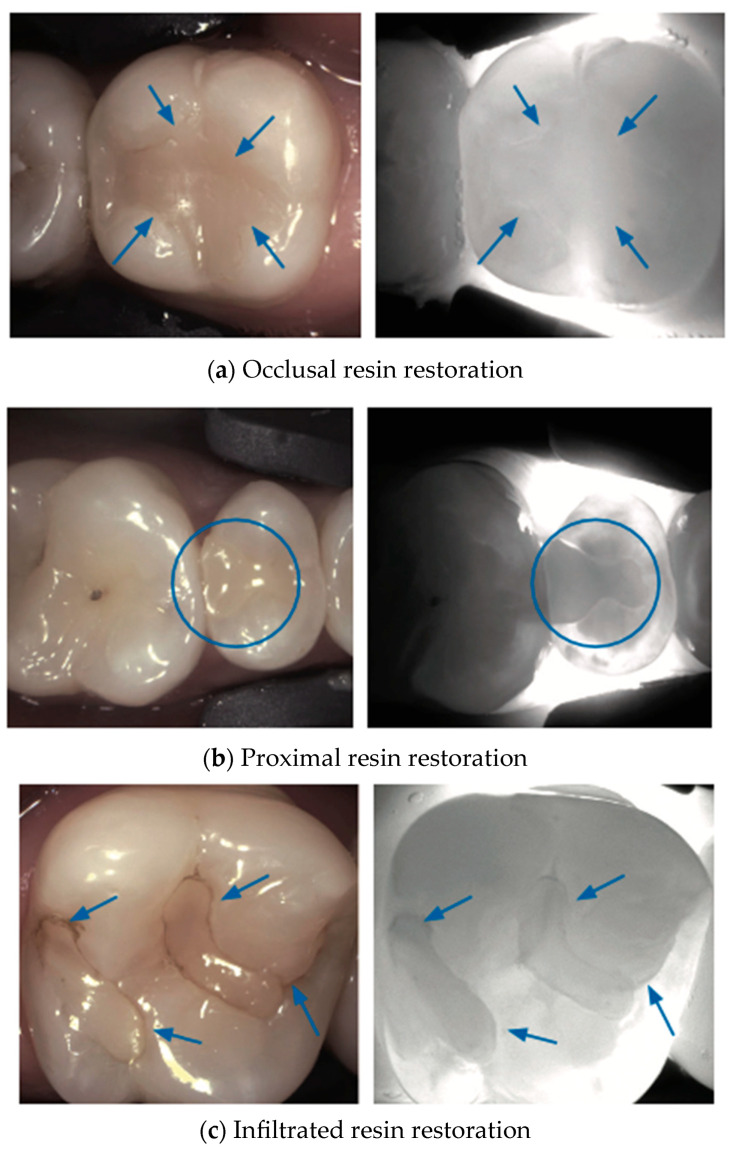
**Occlusal and proximal resin restoration**. Resin restorations on the occlusal surface (**a**) present as areas less translucent than enamel yet blend well with dentin density as they have similar light scattering properties under NIRT (blue arrow). Restorations extending to the proximal surface (**b**) can show marginal differences due to refractive index variations (blue circles). Over time, wear or microleakage can lead to decreased translucency or shadowing of the restoration margins, indicating potential issues such as secondary caries (blue arrows). Composite restorations with discolored or infiltrated margins (**c**) may appear as a high-contrast contoured area (area with a darker contour) under NIRT imaging [17,18,22,25,36].

**Figure 5:**
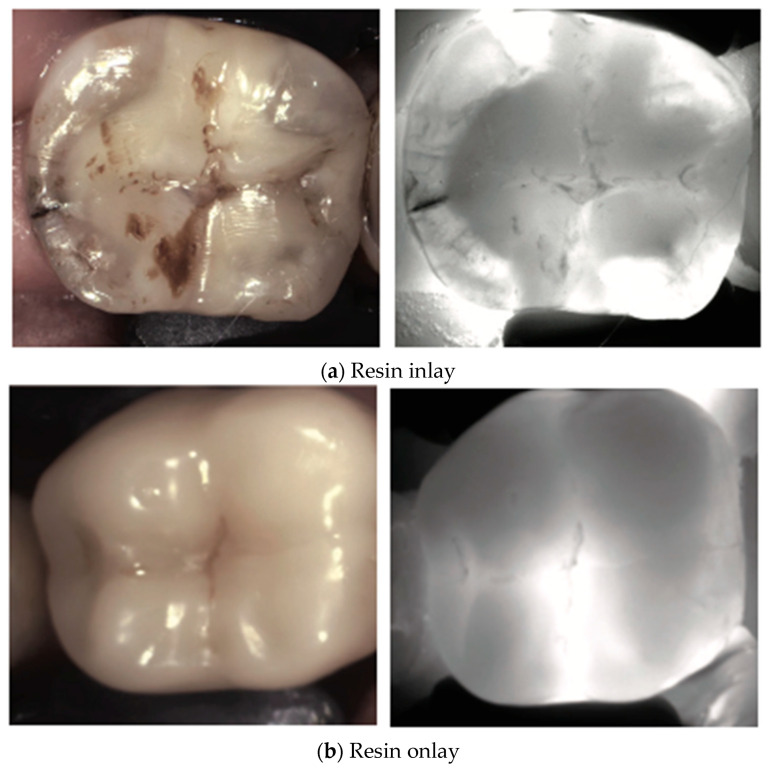
**Resin inlays/onlays**. Inlays (**a**) appear like resin composite restorations. Under NIRT, well-adapted resin onlays (**b**) and inlays typically exhibit a uniform translucency, blending seamlessly with the surrounding tooth structure. However, any marginal discrepancies may appear as areas of altered light transmission, indicating potential discoloration, adaptation issues or secondary caries [13].

**Figure 6:**
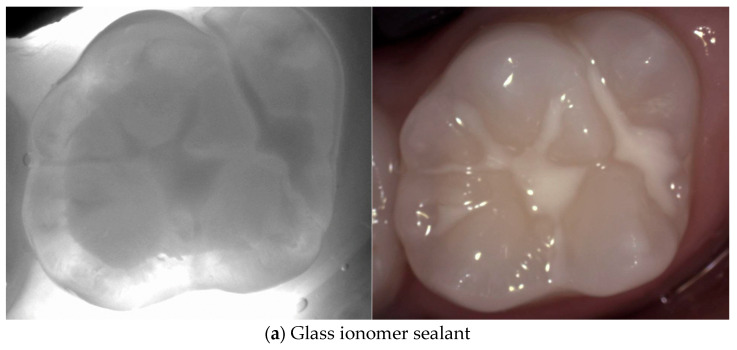

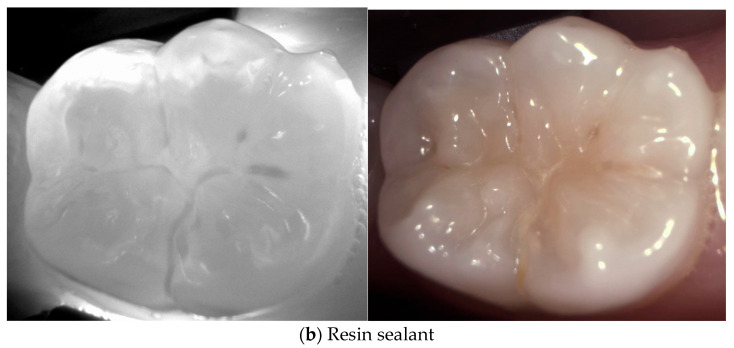
**Sealant of occlusal surfaces**. The image of a sealed tooth depends on the materials used. Glass ionomer sealants (**a**) are opaque which leads to more light scattering and a darker area representing the sealant. Resin sealants (**b**) have a translucency similar to dentin, making them almost invisible if the margin is well polished and integrated. And some resin sealants are completely transparent under NIRT allowing the visualization of the lesion sealed underneath.

**Figure 7:**
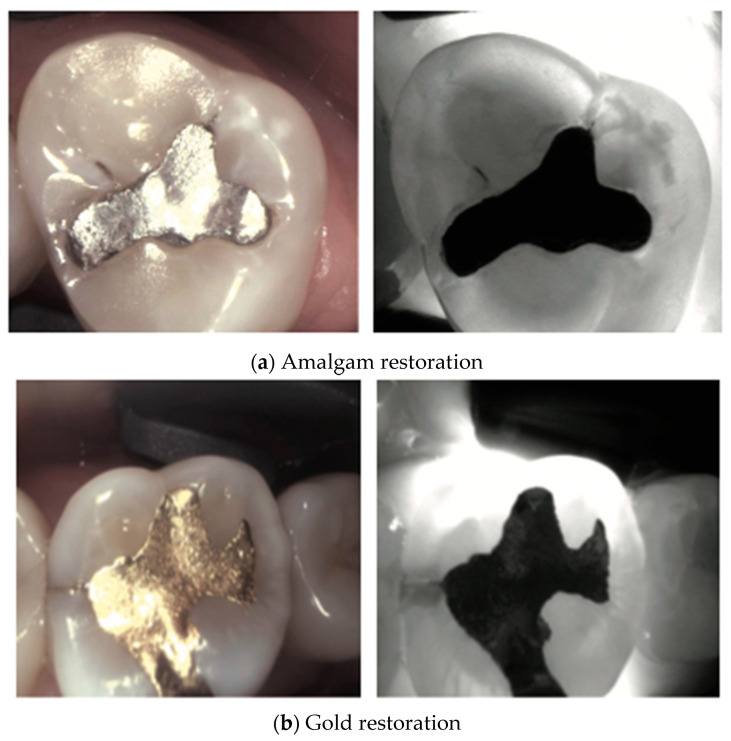

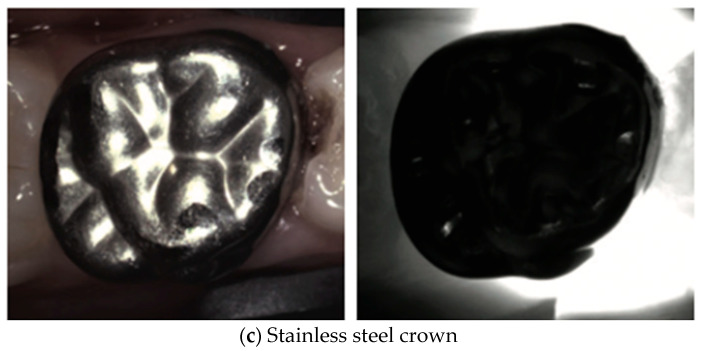
**Metal restorations**. Metal restorations such as amalgams (**a**) or gold (**b**) under NIRT typically appear as opaque regions (black areas) with no light transmission, contrasting with the surrounding tooth structure [37]. However, stainless steel crowns (**c**) under Near-Infrared Transillumination (NIRT) typically appear as fully opaque black teeth with no light penetration.

**Figure 8:**
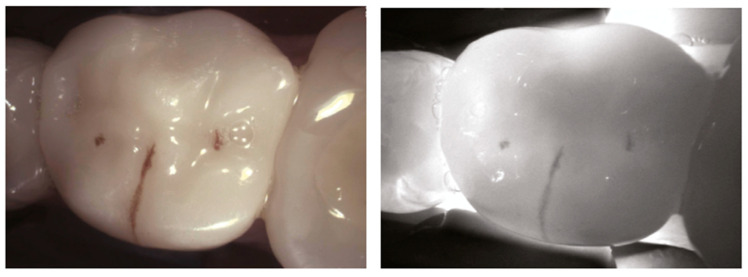
**Ceramic restorations**. Full ceramic crowns under NIRT may appear as homogeneous and slightly translucent structures, while ceramic metallic crowns are more opaque due to the metal base affecting the light scattering.

Various types of dental restorations, such as amalgam, resin composite, and glass ionomers, present distinct interactions with NIRT and affect the transmission and reflection of light, thus influencing their appearance on NIRT images in Figure 4, Figure 5, Figure 6, Figure 7 and Figure 8.

Dental composites have unique spectral signatures in the NIR due to combination absorption bands that can be used for differentiation from tooth structure and other types of composites [34].

Wear or degradation of the restoration may cause localized reduced translucency, revealing potential sealant failure or underlying caries. Advanced sealant failure results in a significant increase in the contrast, indicating compromised integrity of the marginal seal and potential caries development [35].

## 4. Enamel Defects

**Figure 9:**
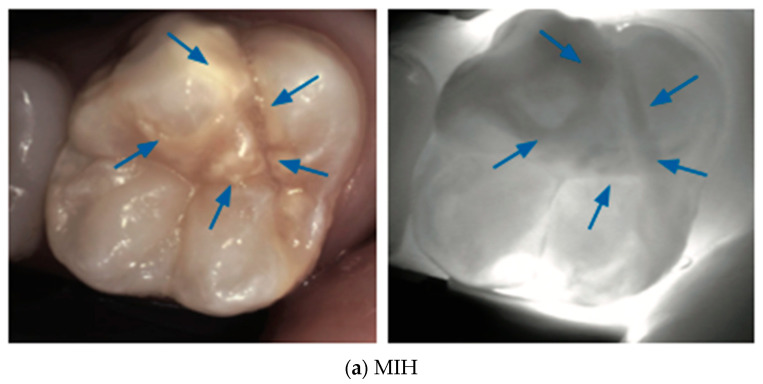

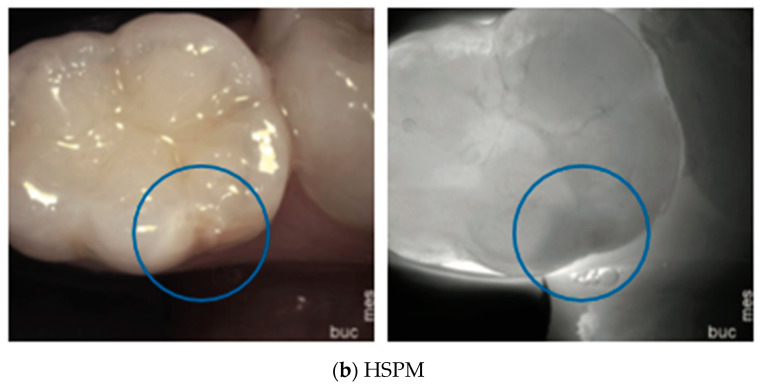
Molar–Incisor Hypomineralization MIH (**a**) and Hypomineralized Second Primary Molars HSPM (**b**): depending on the severity of the defect, affected teeth may exhibit various sizes of localized areas of decreased translucency with various shades of darker gray (blue circle and arrows) under NIRT, indicating hypomineralized enamel regions [39].

**Figure 10:**
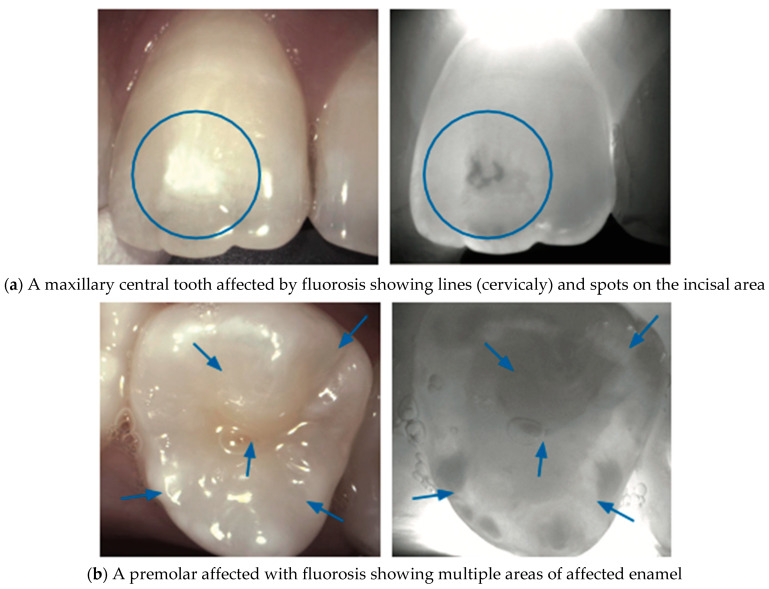
**Fluorosis:** Under NIRT, fluorosis presents varying degrees of enamel opacity, influenced by the extent of mineralization alterations caused by excessive fluoride exposure during tooth development. The distinct appearances of fluorosis lesions under NIRT can help identify the lesions by highlighting areas of hypomineralized enamel or altered mineral content which could appear as lines (**a**) or spots (**b**) (blue circle and arrows). Additionally, NIRT imaging allows differentiation between fluorosis and other enamel defects, contributing to accurate diagnosis and treatment planning [39].

**Figure 11:**
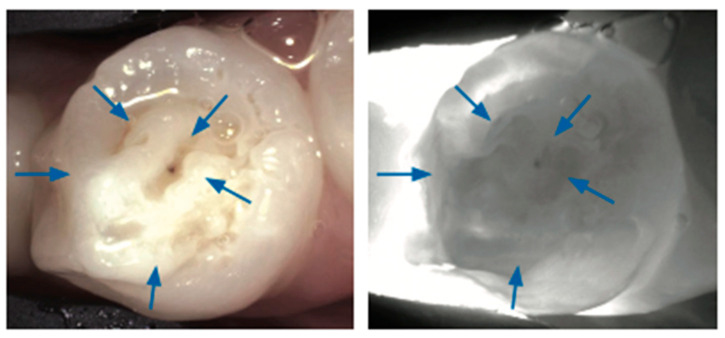
**Hypoplastic enamel**. Hypoplastic enamel defects may present as areas of decreased translucency or altered morphology under NIRT (blue arrows) [40].

**Figure 12:**
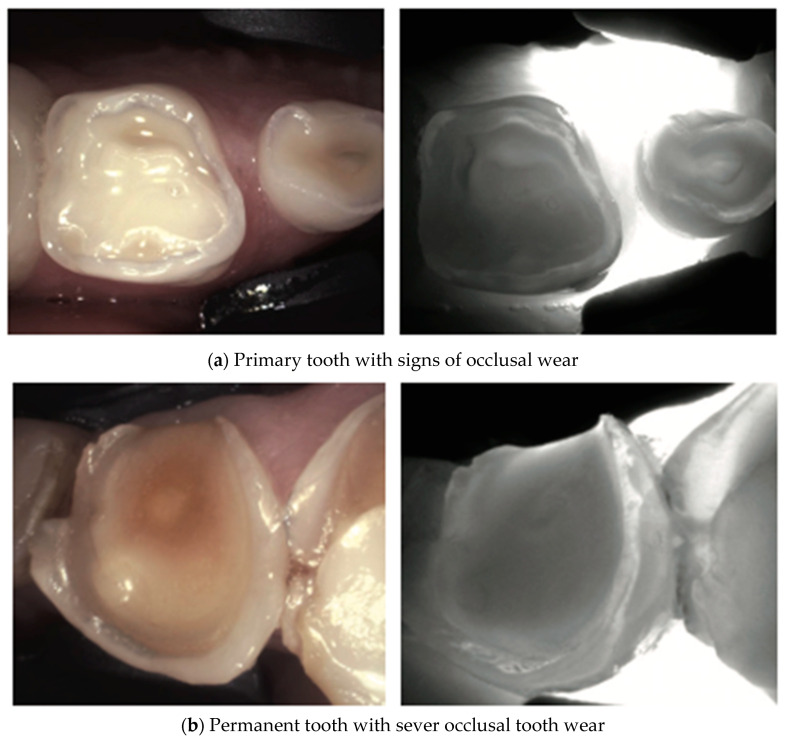
**Tooth wear**. Loss of enamel on the occlusal surface due to attrition or combined attrition and erosion can be seen in primary (**a**) or permanent teeth (**b**). Attrition may be detected using NILT as a large demarcated patch with a dark area as the enamel layer was removed due to wear. Severe loss of occlusal enamel renders the limits of the enamel–dentin junction clearer due to the increased contrast between enamel and dentin. The enamel appears like a radiolucent almost white ring surrounding the EDJ. Sclerotic dentin due to attrition may also appear darker due to the modified structure that can increase the light scattering [38,40].

**Figure 13:**
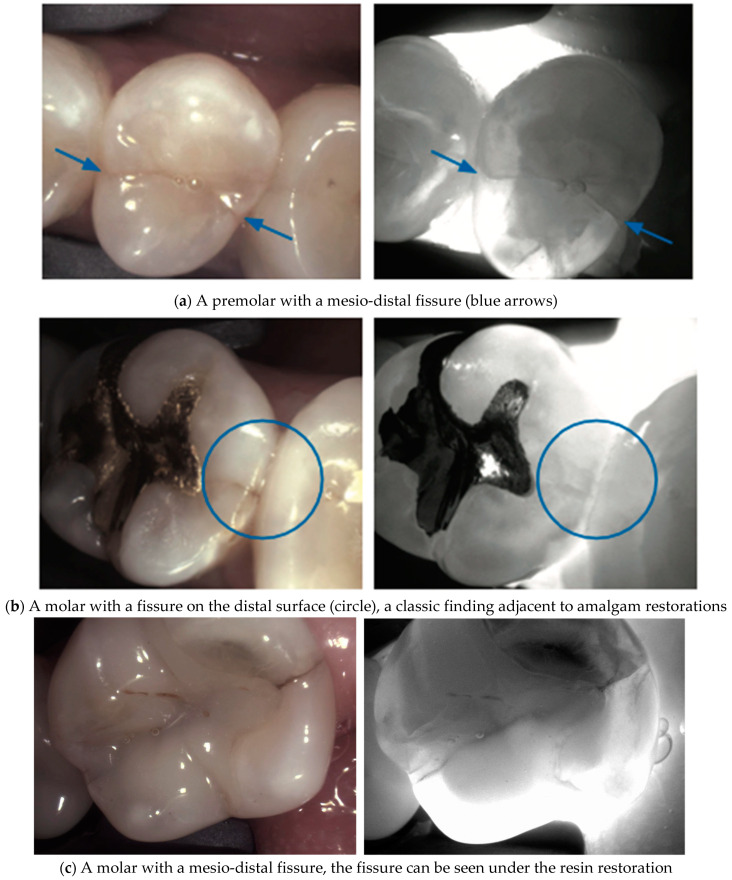
**Fissured and fractured teeth**. Fissured or fractured teeth exhibit disrupted light transmission patterns under NIRT. Cracks and fractures interfere with light propagation in the tooth aiding in crack identification and appearing as a dark line. (**a**,**b**), highlighting areas of the fracture and aiding in diagnosis and treatment planning [40,41]. The fissure can in some cases be visualized and identified under the resin restoration if the material is transparent to near-infrared light (**c**).

The increased porosity of developmental defects causes increased light scattering and increased attenuation in the NIR light; therefore, such defects are easily detected via NIR imaging [38] in Figure 9, Figure 10, Figure 11, Figure 12 and Figure 13.

## 5. Diverse Findings

**Figure 14:**
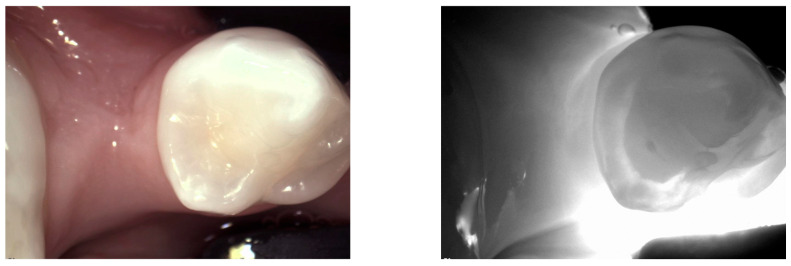
**Missing tooth or spacing**. An edentulous area due to a lost tooth or a spacing between teeth may appear as a smooth grey area reflecting the absence of dental structures and allowing visualization of the gingiva under NIRT. Depending on the space between the teeth and the thickness of the dental ridge, the translucency may vary due to the light scattering in the different tissues.

**Figure 15:**
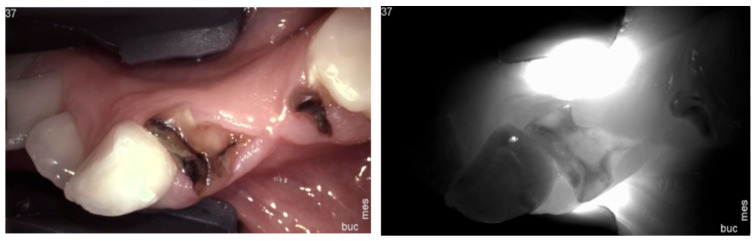
**Retained root fragments**. If the fragments are supragingival, under NIRT, they may appear as a shadow or a darker area due to their denser structure compared to the surrounding gingiva.

**Figure 16:**
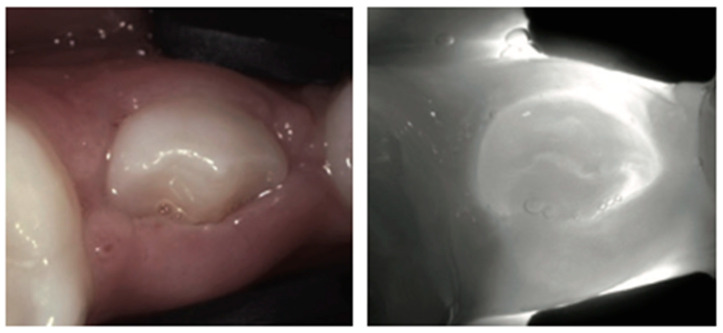
**Erupting tooth**. Under NIRT, an erupting tooth may present as a structure of altered translucency surrounded by gingival tissues.

**Figure 17:**
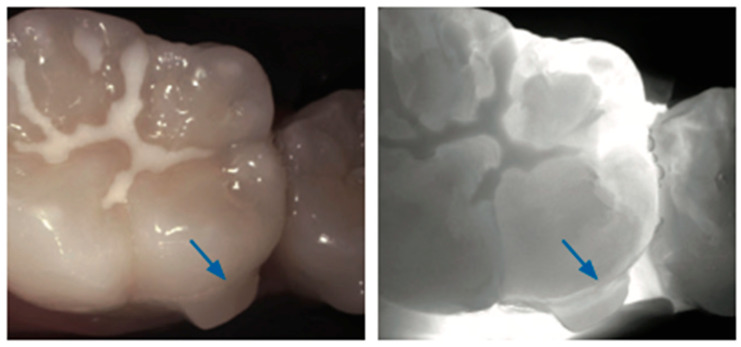
**Invisalign attachments**. Typically made of tooth-colored resin composite materials (arrow), they may appear as structures with similar translucency to dental tissue under NIRT. Their presence may be detectable as areas of altered light transmission compared to the surrounding enamel [40].

**Figure 18:**
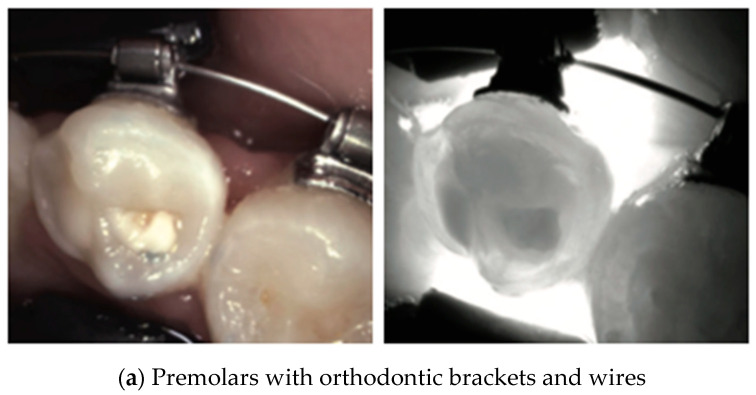

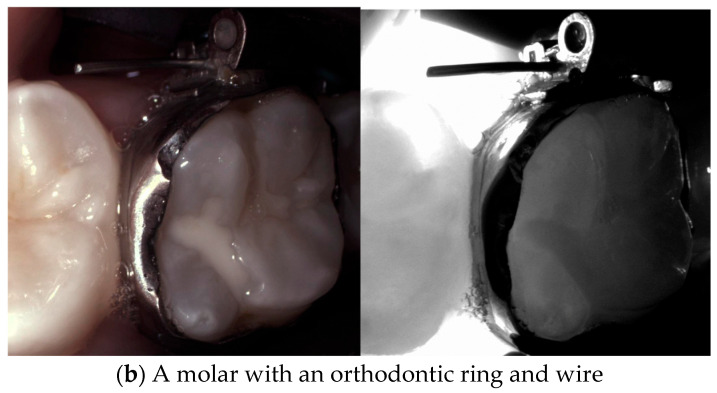
**Orthodontic brackets**. Orthodontic brackets (**a**) and rings (**b**) are made of metal in most cases and they appear under NIRT as black opaque structures on tooth surfaces. The use of NIRT for the detection of early proximal caries while undergoing an orthodontic treatment is one of its valuable advantages [40]. Thus, NIRT can also be used to monitor the efficacy of preventive measures like fluoride application in preventing demineralizing through the course of treatment.

**Figure 19:**
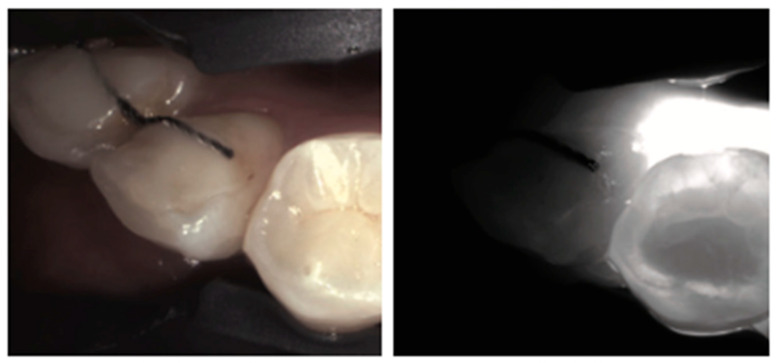
**Orthodontic retainer wires**. Under NIRT they will appear as thin, dark lines crossing tooth surfaces. This observation can provide incidental information about past orthodontic treatment.

**Figure 20:**
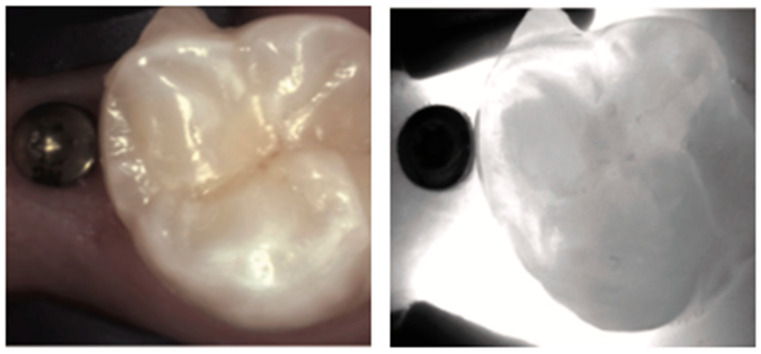
**Implant protection screws**. Like other metallic structures, under NIRT, an implant cover will appear as a distinct, black opaque round structure.

Clinical and X-ray examinations may detect various incidental findings. Similarly, incidental findings (such as various dental structures and appliances (Figure 14, Figure 15, Figure 16, Figure 17, Figure 18, Figure 19 and Figure 20)) detected during an NIRT examination can demonstrate the wide-ranging diagnostic capabilities of NIRT that could be very valuable for other purposes such as forensic odontology. Dental identification of a deceased person is a primary utility of forensic odontology. The comparison of a missing person’s ante-mortem dental records (i.e., written data, dental casts, photographs and digital images, and radiographs) with the post-mortem dental evidence from unknown human remains has long been recognized as one of the most reliable means of human identification [42]. The literature has demonstrated that intraoral photographs stand as the best imaging approach for the registration of clinically detectable dental identifiers for human dental identification and they strongly endorse the routine use of oral photography in daily dental practice [43]. In forensic identification of a deceased person, recognition of any details such as the type of material and the fluorescence and reflectance value of a tooth filling over and above the conventional dental descriptors of restored, non-restored, missing, and decayed teeth offer one more degree of certainty to examiners [44]. Post-mortem NIRT images could also be a valuable tool for forensic odontologists for human identification when combined with clinical images. This paper presents a pioneering work connecting NIRT imaging with the field of dental forensic identification. 

## 6. Conclusions

This Atlas of Near-Infrared Transillumination Images of human teeth has been developed for dental healthcare professionals such as dentists, hygienists, dental students, and forensic odontologists. This document provides a valuable resource for teaching activities in dental training and continuing education. This Atlas compiles the most frequent and useful dental features and restorative materials on NIRT images that are of interest to clinicians.

## Data Availability

The data presented in this study are available upon request from the corresponding authors.
